# The Scavenging Activity of Coenzyme Q_10_ Plus a Nutritional Complex on Human Retinal Pigment Epithelial Cells

**DOI:** 10.3390/ijms25158070

**Published:** 2024-07-24

**Authors:** Maria Hernandez, Sergio Recalde, Jaione Bezunartea, Maite Moreno-Orduña, Idoia Belza, Ainara Chas-Prat, Elena Perugini, Alfredo Garcia-Layana, Patricia Fernández-Robredo

**Affiliations:** 1 Retinal Pathologies and New Therapies Group, Experimental Ophthalmology Laboratory, Department of Ophthalmology, Clinica Universidad de Navarra, Navarra Institute for Health Research, IdiSNA, (RICORS-TERAV), 31008 Pamplona, Spain; mahersan@unav.es (M.H.); srecalde@unav.es (S.R.); jbezunartea@unav.es (J.B.); achas@external.unav.es (A.C.-P.); aglayana@unav.es (A.G.-L.); 2 Retinal Pathologies and New Therapies Group, Experimental Ophthalmology Laboratory, Department of Ophthalmology, Clinica Universidad de Navarra, 31008 Pamplona, Spain; maimoreno@unav.es (M.M.-O.); idoiabelza@unav.es (I.B.); eperugini@alumni.unav.es (E.P.)

**Keywords:** age-related macular degeneration (AMD), diabetic retinopathy (DR), coenzyme Q_10_, oxidative stress, mitochondrial stress, ARPE-19, DRP-1, caspase-3

## Abstract

Age-related macular degeneration (AMD) and diabetic retinopathy (DR) are common retinal diseases responsible for most blindness in working-age and elderly populations. Oxidative stress and mitochondrial dysfunction play roles in these pathogenesis, and new therapies counteracting these contributors could be of great interest. Some molecules, like coenzyme Q_10_ (CoQ_10_), are considered beneficial to maintain mitochondrial homeostasis and contribute to the prevention of cellular apoptosis. We investigated the impact of adding CoQ_10_ (Q) to a nutritional antioxidant complex (Nutrof Total^®^; N) on the mitochondrial status and apoptosis in an in vitro hydrogen peroxide (H_2_O_2_)-induced oxidative stress model in human retinal pigment epithelium (RPE) cells. H_2_O_2_ significantly increased 8-OHdG levels (*p* < 0.05), caspase-3 (*p* < 0.0001) and TUNEL intensity (*p* < 0.01), and RANTES (*p* < 0.05), caspase-1 (*p* < 0.05), superoxide (*p* < 0.05), and DRP-1 (*p* < 0.05) levels, and also decreased *IL1β*, *SOD2*, and *CAT* gene expression (*p* < 0.05) vs. control. Remarkably, Q showed a significant recovery in *IL1β* gene expression, TUNEL, TNFα, caspase-1, and JC-1 (*p* < 0.05) vs. H_2_O_2,_ and NQ showed a synergist effect in caspase-3 (*p* < 0.01), TUNEL (*p* < 0.0001), mtDNA, and DRP-1 (*p* < 0.05). Our results showed that CoQ_10_ supplementation is effective in restoring/preventing apoptosis and mitochondrial stress-related damage, suggesting that it could be a valid strategy in degenerative processes such as AMD or DR.

## 1. Introduction

Oxidative stress and mitochondrial dysfunction are involved in the pathogenesis of age-related macular degeneration (AMD) and diabetic retinopathy (DR) [1,2,3,4]. Both are complex eye disorders with multifactorial etiologies and many factors have been implicated in their pathogenesis and progression, including oxidative damage, inflammation, aging, genetic predisposition, and environmental influences. AMD is characterized by retinal pigmented epithelium (RPE) dysfunction and damage to Bruch’s membrane and the choriocapillaris complex [5], and DR is a microvascular disease characterized by blood flow alterations, pericyte loss, the downregulation of endothelial cells, tight junctions, and the thickening of the basement membrane [6,7].

Mitochondria dynamics are affected by several stressors, like oxidative stress, provoking an imbalance in its fission/fusion processes [8]. Mitochondrial fission creates new mitochondria during cell division and facilitates the segregation of damaged mitochondria, whereas mitochondrial fusion enables the exchange of intramitochondrial material between mitochondria. The balance between fission/fusion processes determines the mitochondrial morphology and adapts it to the cellular metabolic requirements [9]. Exorbitant mitochondrial fission, resulting in mitochondrial disintegration or fragmentation, may be a consequence of oxidative stress in neurodegenerative disorders [9].

Coenzyme Q_10_ (CoQ_10,_) is a fat-soluble quinone involved in the mitochondrial respiratory chain, synthetized mainly in the inner membrane of the mitochondria and secondarily in the endoplasmic reticulum Golgi apparatus [10], and exerts protective roles in various metabolic, antioxidant, and inflammatory [11] and ferroptosis processes [12]. CoQ_10_ plays an essential role in the normal function of the electron transport chain and has been reported to exhibit neuroprotective activity in a range of disorders, including cerebral ischemia [13] instead of Parkinson’s disease and Huntington’s disease [14]. Usually, its expression decreases with age and is therefore correlated with degenerative diseases such as AMD [15]. Lower plasma levels than in the controls were observed in AMD and DR patients [15,16,17]. The lack of protection provided by CoQ_10_ could affect the development of AMD and DR. Therefore, CoQ_10_ has been extensively utilized for food supplements and as a dietary supplement that is very important for maintaining human health. 

This study aimed to elucidate the effect of adding CoQ_10_ to a nutritional antioxidant complex, Nutrof total^®^, in an adult RPE cell line (ARPE-19) subjected to oxidative stress. We focused on its effect on apoptosis, cytokines release, and DNA oxidative damage, especially that related to the mitochondria. Therefore, we evaluated the mitochondrial function under oxidative stress conditions. We analyzed specifically the dynamin-related protein (DRP1), a protein that physiologically serves to eliminate damaged mitochondria during fission [18], mitochondrial DNA quantification, mitochondrial superoxide concentrations and mitochondrial membrane potential (mtΔψ) in live cells. 

## 2. Results

### 2.1. CoQ_10_ Plus N Restored Oxidative Stress-Related DNA Damage 

**Figure 1:**
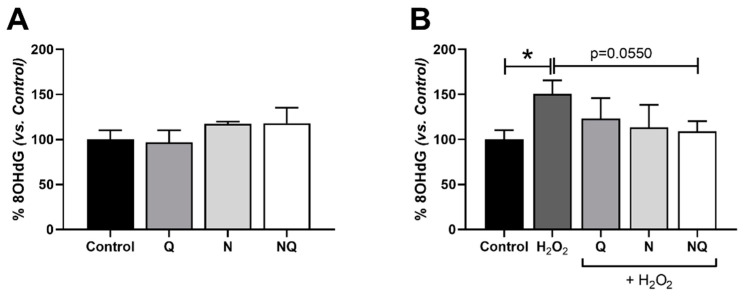
DNA oxidative damage analyzed as 8-OHdG levels in ARPE-19 cells’ supernatants by ELISA in basal conditions (**A**) and after the addition of H_2_O_2_ (600 µM, 1 h) and antioxidant treatments in concomitance for 30 min (**B**) (* *p* < 0.05 vs. control) (*n* = 3). The application of NQ showed a tendency to significantly reduce 8-OHdG levels vs. H_2_O_2_ control group (*p* = 0.0550).

Under basal conditions, a similar response in 8-hydroxy-2’-deoxyguanosine (8-OHdG) levels was observed in treated groups with different antioxidants (*n* = 3). Although a slight increase is observed in N and NQ groups, this did not reach statistical significance (Figure 1A). Oxidative stress induced by H_2_O_2_ revealed a statistically significant increase in DNA damage (*p* < 0.05, Figure 1B). Under an oxidant environment, all treatments were able to reduce 8-OHdG levels, although the reduction was only nearly significant in the NQ group (*p* = 0.055, Figure 1B).

### 2.2. CoQ_10_ Plus N Protects from Early and Late Apoptosis Induced by Oxidative Stress 

**Figure 2:**
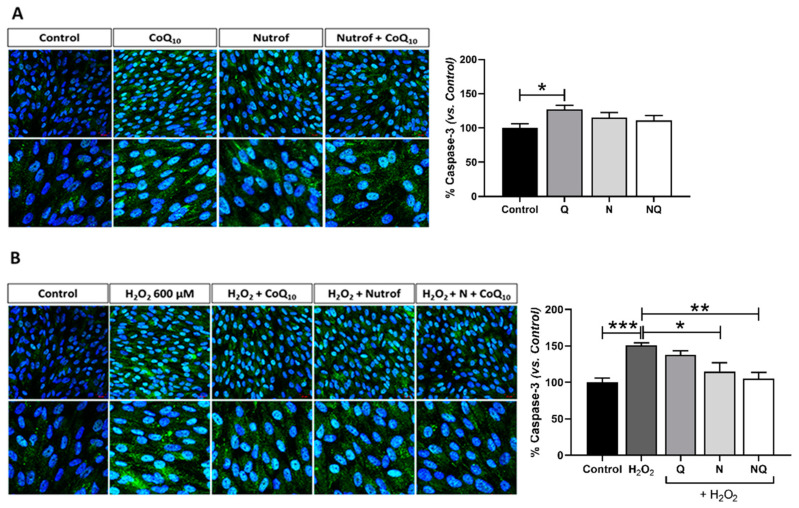
Percentage of the fluorescence intensity of caspase-3 (green) immunolabeling in basal conditions after Q, N, and NQ showed statistical differences between control and Q (*p* < 0.05) (**A**). Oxidative environment induced by H_2_O_2_ increased caspase-3 immunofluorescence vs. control group (*p* < 0.001) (**B**) (*n* = 3). After N and NQ with oxidative stress, significant differences were observed vs. H_2_O_2_ group (* *p* < 0.05, ** *p* < 0.01, *** *p* < 0.001). Nuclei were labeled with 4′,6-diamidino-2-phenylindole (DAPI, blue). Scale bar: 20 µm.

Early apoptosis was analyzed and quantified by active caspase-3 immunofluorescence on ARPE-19 cells after several conditions of H_2_O_2_ (Figure S1A) to select the appropriate concentration and incubation time (*n* = 3). Basal conditions (Figure 2A) and antioxidant treatments with induced oxidative stress (600 µM H_2_O_2_ for 3 h) (Figure 2B) were analyzed. Under basal standard conditions, caspase-3 immunofluorescence revealed that there is a similar fluorescence signal intensity in treated groups with antioxidants, except for the Q group which showed a statistically significant increase when compared to the control (*p* < 0.05, Figure 2A). The oxidative environment induced by H_2_O_2_ revealed a statistically significant increase in caspase-3 expression (*p* < 0.001, Figure 2B). N and NQ treatments in concomitance with H_2_O_2_ were able to significantly reduce early apoptosis induction when compared to the H_2_O_2_ control (*p* < 0.05 and *p* < 0.01, respectively, Figure 2B). The Q group did not show any effect on early apoptosis under the conditions used. 

**Figure 3:**
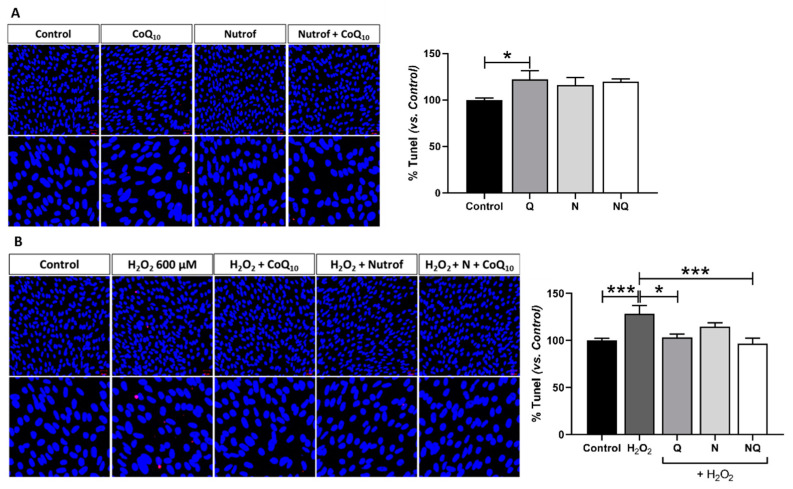
Percentage of TUNEL fluorescence intensity (red) in basal conditions after Q, N, and NQ showed statistical differences between control and Q (* *p* < 0.05) (**A**). H_2_O_2_ group showed a significant increase vs. control group (*** *p* < 0.001). After Q, N, and NQ treatments in concomitance with oxidative stress, a significant reduction was observed in Q and NQ vs. H_2_O_2_ group (* *p* < 0.05 and *** *p* < 0.001) (**B**) (*n* = 3). Nuclei were labeled with 4′,6-diamidino-2-phenylindole (DAPI, blue). Scale bar: 20 µm.

Furthermore, we analyzed DNA fragmentation by TUNEL in order to study the late stage of apoptosis. Under basal conditions (Figure 3A), similarly to the early apoptosis results, TUNEL revealed no changes in the fluorescence signal intensity in treated groups with antioxidants when compared to the control, except for the Q group which showed a statistically significant increase (*p* < 0.05, Figure 3A) (*n* = 3). Oxidative stress induction demonstrated an increase in the late apoptosis signal according to the experimental design showed in Table S1 (*p* < 0.001, Figure 3B). Concomitant treatment with either N, Q, or NQ were able to restore the oxidative damage (Figure 3B). Q and NQ treatment additions were able to induce a statistically significant reduction in the TUNEL signal when compared to H_2_O_2_ (*p* < 0.05, *p* < 0.001, Figure 3B). In contrast, although N was able to reduce the TUNEL signal, this difference was not statistically significant when compared to the H_2_O_2_ group (Figure 3B).

### 2.3. CoQ_10_ Reduces Caspase-1 Levels Increased by Oxidative Stress

**Figure 4:**
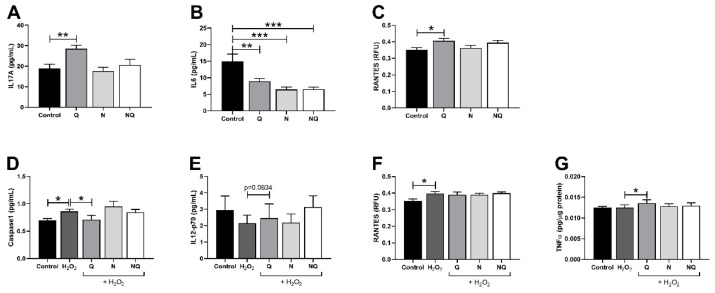
Quantification of cytokine levels in which changes have been observed in standard conditions and under oxidative stress with treatments Q, N, and NQ (*n* = 4). Levels of IL17A, IL6, and RANTES in ARPE-19 cells supernatant (**A**–**C**) in standard conditions. Levels of caspase-1, IL12-p70, and RANTES in ARPE-19 cells supernatant after oxidative stress conditions (**D**–**F**) and TNFα levels in lysates after oxidative stress conditions (**G**). Lysates’ data are presented as pg/µg protein and supernatants’ data are presented as pg/mL. RANTES data are presented as RFU. For all data mean ± SEM are presented. * *p* < 0.05, ** *p* < 0.01, and *** *p* < 0.001 vs. H_2_O_2_. Q—coenzyme Q_10_, N—Nutrof total, NQ—Nutrof total + CoQ_10_.

ARPE-19 cells’ supernatants and lysates of caspase-1, IL12-p70, IL17A, IL18, IL1β, IL6, RANTES, and TNFα were analyzed to determine intracellular levels (*n* = 4). Under standard conditions, the addition of treatments did not modify the levels of caspase-1, IL12-p70, IL17A, IL18, IL1β, IL6, TNFα, and RANTES (Figure S2) in ARPE-19 lysates. Released cytokines were also similar in the treatment groups when compared to the control (Figure S2), except for IL17A and RANTES, which showed an increase in the Q group when compared to the control (*p* < 0.01 and *p* < 0.05, respectively; Figure 4A,C). IL6 released levels were significantly reduced in the Q, N, and NQ treatments (*p* < 0.01, *p* < 0.01 and *p* < 0.001, respectively; Figure 4B). 

Oxidative stress induction significantly increased the caspase-1 and RANTES levels vs. the control group (Figure 4D and Figure 4F, respectively, *p* < 0.05). Only caspase-1 levels were significantly reduced after the Q addition when compared to the H_2_O_2_ group (Figure 4D, *p* < 0.05). However, N and NQ were not able to modify the cytokines levels (Figure 4D–G).

### 2.4. Interleukin (IL) 1β, Superoxide Dismutase 2 (SOD2) and Catalase (CAT) Gene Expression

**Figure 5:**
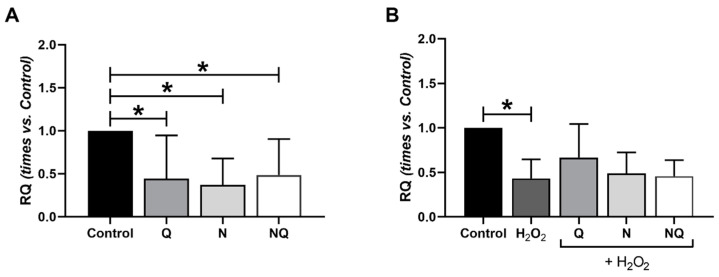
Quantification of *SOD2* gene expression of cultured ARPE-19 cells in standard conditions and under oxidative stress with Q, N, and NQ treatments (*n* = 4). *SOD2* expression in standard conditions showed a significant reduction with all antioxidant treatments (**A**). H_2_O_2_ group showed a significant decrease vs. control group (* *p* < 0.05). *SOD2* expression in ARPE-19 cells with 2 h of H_2_O_2_ in concomitance showed no significant reduction with treatments (**B**). For all data, mean ± SEM are presented. * *p* < 0.05 vs H_2_O_2_ group. Q—coenzyme Q_10_, N—Nutrof total, NQ—Nutrof total + CoQ_10_.

Oxidative stress induction with H_2_O_2_ for 2 h produced a decrease in *SOD2* expression in both timepoints when compared to the control group, although it was significant only at 2 h (*p* < 0.05, Figure S4A) (*n* = 4). Under basal conditions, all antioxidant treatments (Q, N, and NQ) showed a significant reduction in *SOD2* expression with respect to the control (*p* < 0.05, Figure 5A). Antioxidant treatment (30 min) concomitance with H_2_O_2_ (1 h induction) provoked a significant decrease in *SOD2* expression when compared to the control (*p* < 0.05, Figure S4B,C). After 2 h of oxidative damage with H_2_O_2,_ a significant reduction in *SOD2* gene expression was observed when compared to control group (Figure 5B, *p* < 0.05); however, the Q, N, and NQ treatments did not restore the effect, although there is a tendency for this to increase under oxidative conditions (Figure 5B).

**Figure 6:**
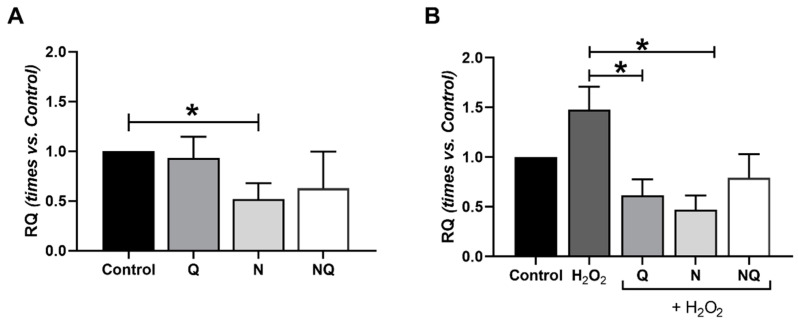
Quantification of *ILβ1* expression of cultured ARPE-19 cells in standard conditions and under oxidative stress with treatments Q, N and NQ (*n* = 4). *ILβ1* expression significantly decreased with N antioxidant treatment * *p* < 0.05 vs. control (**A**). *ILβ1* expression in ARPE-19 cells with 1 h of H_2_O_2_ in concomitance decreased after Q and N treatment (**B**) (* *p* < 0.05) vs. H_2_O_2_ group. Q—coenzyme Q_10_, N—Nutrof total, NQ—Nutrof total + CoQ_10_.

Figure 6 shows the results obtained in the comparative quantification of *ILβ1* expression (*n* = 4). After 2 h of damage with H_2_O_2_, a very significant decrease in *ILβ1* expression was observed (*p* < 0.01, Figure S4C), and a non-significant increase was observed after 1 h of damage (Figure S4C). Under the basal conditions, treatments showed an effect of decreasing *ILβ1* expression which was only significant for the N group vs. the control (*p* < 0.05, Figure 6A). After the administration of the antioxidant treatments in concomitance with H_2_O_2_ (1 h), the Q and N groups were able to significantly decrease *ILβ1* expression vs. the H_2_O_2_ group (*p* < 0.05, Figure 6B). After 2 h of oxidative damage, no changes were observed for all groups (*p* < 0.01, Figure S4D).

**Figure 7:**
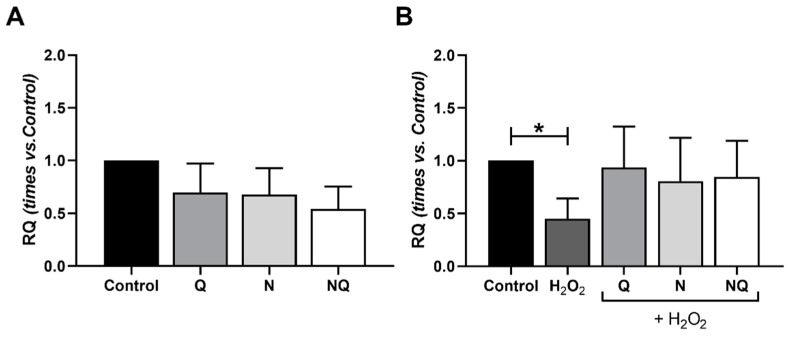
Quantification of *CAT* expression of cultured ARPE-19 cells in standard conditions and under oxidative stress with treatments Q, N, and NQ (*n* = 4). No changes were observed in *CAT* expression in basal conditions with antioxidant treatments (**A**). *CAT* expression in ARPE-19 cells with 1 h of H_2_O_2_ concomitance showed a decrease only in H_2_O_2_ group vs. control (* *p* < 0.05) (**B**). Q—coenzyme Q_10_, N—Nutrof total, NQ—Nutrof total + CoQ_10_.

After 1 h of damage with H_2_O_2_, a statistically significant decrease in *CAT* expression and a non-significant increase was observed after 2 h of damage (*p* < 0.05, Figure S4E). Figure 7 shows the results obtained for *CAT* gene expression (*n* = 4). Under basal conditions, treatments did not show a statistically significant modification (Figure 7A). When used in concomitance with H_2_O_2_, all treatments showed a stabilizing effect against the alterations observed with oxidative stress, maintaining similar *CAT* gene expression values as the control group for both timepoints (Figure S4F and Figure 7B). 

### 2.5. Mitochondrial Dysfunctionality and Damaged Mitochondrial DNA (mtDNA)

#### 2.5.1. Mitochondrial Superoxide Production

**Figure 8:**
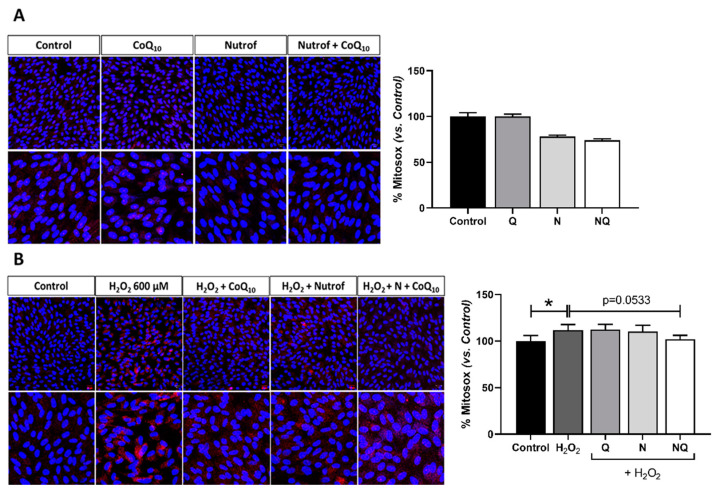
Percentage of mitochondrial superoxide indicator in live ARPE-19 cells measured by MitoSOX (red) in standard conditions and under oxidative stress with treatments Q, N, and NQ (*n* = 3). No changes in basal conditions were observed (**A**). H_2_O_2_ group showed a significant increase vs. control group (* *p* < 0.05), (**B**) and after H_2_O_2_ in concomitance, only the NQ treatment decreased MitoSOX (*p* = 0.0533) (B). Q—coenzyme Q_10_, N—Nutrof total, NQ—Nutrof total + CoQ_10_. * *p* < 0.05. Nuclei were labeled with 4′,6-diamidino-2-phenylindole (DAPI) (blue). Scale bar: 20 µm.

A mitochondrial superoxide indicator was detected using the fluorescent assay MitoSOX in live ARPE-19 cells. The dose selected to be used in the subsequent analysis was 600 µM after 2 h (Figure S5) (*n* = 3). In basal conditions, a decrease in superoxide levels was observed in N and NQ groups; however, it did not reach statistical significance (Figure 8A). The oxidative environment induced by H_2_O_2_ showed a statistically significant increase in superoxide quantification when compared to the control group (*p* < 0.05, Figure 8B), and only the NQ treatment was able to reduce its levels, although the reduction was not statistically significant (*p* = 0.053; Figure 8B).

#### 2.5.2. Mitochondrial DNA (mtDNA) Amount

**Figure 9:**
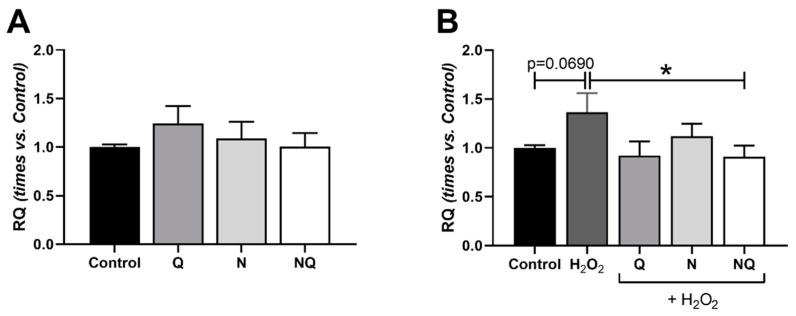
Mitochondrial DNA amount of cultured ARPE-19 cells measured by *12S* RT-PCR under standard conditions and under oxidative stress with treatments Q, N, and NQ (*n* = 4). No changes were observed in the mitochondrial DNA amount in cells treated with different treatments under basal conditions (**A**). H_2_O_2_ group showed an almost significant increase vs. the control group (*p* = 0.0690) (**B**) and the NQ group in concomitance with H_2_O_2_ significantly decreased mtDNA vs. the H_2_O_2_ group * *p* < 0.05 (**B**). Q—coenzyme Q_10_, N—Nutrof total, NQ—Nutrof total + CoQ_10_.

Mitochondrial DNA was measured under basal conditions with antioxidant treatments (Q, N, and NQ), and no significant differences were found when compared with the control group (Figure 9A) (*n* = 4). Under oxidative stress induction with H_2_O_2_, an increase in the amount of mtDNA in the group treated only with H_2_O_2_ was observed, with differences close to significance (*p* = 0.069) vs. the control group (Figure 9B) (*n* = 4). Q and NQ treatments were able to reduce the amount of mtDNA generated by oxidative stress conditions to values similar to the control group, being statistically significant in the case of the NQ group when compared to the H_2_O_2_ group (*p* < 0.05, Figure 9B). 

#### 2.5.3. CoQ_10_ Decreases Mitochondrial Membrane Potential (mtΔψ) under Oxidative Stress Conditions

**Figure 10:**
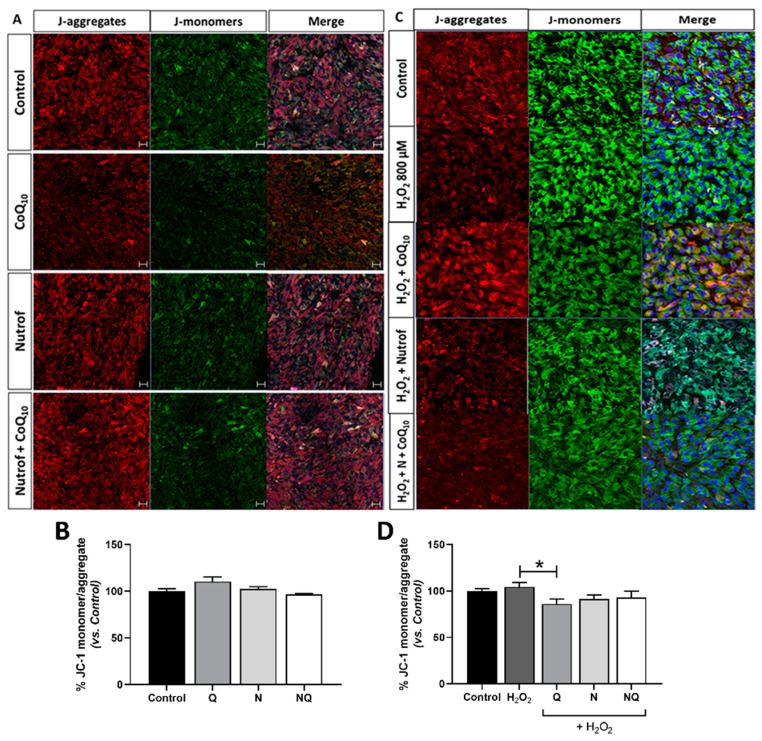
Mitochondrial membrane potential (mtΔψ) determined by live JC-1 measurement in ARPE-19 cells under basal conditions (**A**,**B**) and in concomitance with oxidative stress conditions with antioxidants treatments (**C**,**D**) (*n* = 3). J-monomers, green; J-aggregates, red. No changes were observed in JC-1 under basal conditions (**A**); however, in concomitance with H_2_O_2_ only, the Q treatment significantly decreased the mtΔψ vs. H_2_O_2_ group (**B**) (* *p* < 0.05). Q—coenzyme Q_10_, N—Nutrof total, NQ—Nutrof total + CoQ_10_. Nuclei were labeled with 4′,6-diamidino-2-phenylindole (DAPI) (blue). Scale bar: 20 µm.

Under basal conditions, the JC-1 ratio was slightly increased in the Q group vs. the control. N and NQ groups showed a similar value when compared to the control (Figure 10A,B) (*n* = 3). After oxidative stress induction, an increase in the JC-1 ratio was observed when compared to the control group, which was not statistically significant. A statistically significant reduction in JC-1 was observed in the Q group compared to H_2_O_2_ (* *p* < 0.05). The N and NQ groups showed a similar value when compared to control (Figure 10C,D).

#### 2.5.4. Mitochondrial Dysfunction Determined by DRP-1 Immunofluorescence

**Figure 11:**
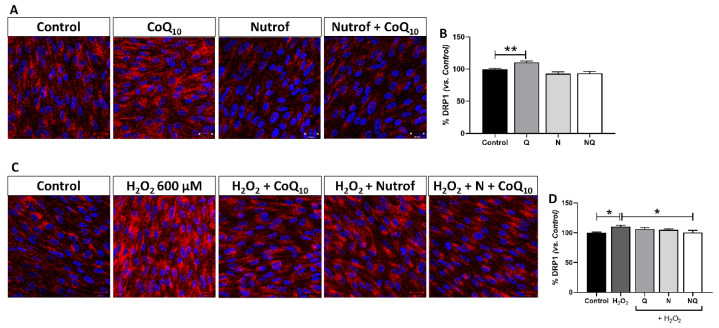
Percentage of mitochondrial DRP-1 (red) measurement in ARPE-19 cells under basal conditions (**A**,**B**) and under oxidative stress conditions with treatments Q, N, and NQ (**C**,**D**) (*n* = 4). Q treatment significant increased DRP-1 under basal conditions (**B**) (** *p* < 0.01). H_2_O_2_ group showed a significant increase vs. control group (* *p*< 0.05) (**B**). After concomitance with H_2_O_2,_ only NQ treatment showed a significant decrease vs. H_2_O_2_ group (* *p* < 0.05). Q—coenzyme Q_10_, N—Nutrof total, NQ—Nutrof total + CoQ_10_. Nuclei were labeled with 4′,6-diamidino-2-phenylindole (DAPI) (blue). Scale bar: 20 µm.

Under basal conditions DRP-1 showed a similar fluorescence signal intensity in treated groups compared to the control group, except for the Q group which exhibited a statistically significant increase when compared to the control group (*n* = 4) (*p* < 0.01, Figure 11A,B). The oxidative environment induced by H_2_O_2_ revealed a statistically significant increase in DRP-1 fluorescence intensity quantification when compared to the control (*p* < 0.05, Figure 11C,D). Under the oxidative environment, treatments were able to reduce DRP-1 levels, which was statistically significant only for the NQ group when compared to H_2_O_2_ (*p* < 0.05, Figure 11D).

## 3. Discussion

This study demonstrates that adding either CoQ_10_ or a nutritional complex (Nutrof total^®^), or the complex in combination with CoQ_10_, can reverse the cellular damage induced by oxidative stress in human RPE cells in vitro. The main mechanisms through which the combined supplementation exerts RPE protection seem to relate to its antioxidant activity, its ability to reduce apoptosis, and its ability to stabilize mitochondrial parameters. 

In previous studies from our group, a synergistic antioxidant and anti-inflammatory effect of Nutrof total along with vitamin D in ARPE-19 cells was also described [19]. Hundreds of papers on antioxidant synergism have been published so far, but the majority of them do not elucidate the mechanism of the synergistic activity [20,21].

CoQ_10_ is a molecule that possesses antioxidant, anti-inflammatory, and neuroprotective properties in some retinal neurodegenerative and ocular diseases [22]. In AMD and RP pathology, RPE cells and retinal endothelial cells undergo several subcellular accumulated damages, such as an increase in lesions in DNA [23,24,25], mitochondrial DNA degradation [26,27], cellular apoptosis [28,29], inflammation [30,31], and mitochondrial dysfunction [2,32] which contribute to the onset of the disease. All these events are strongly correlated with oxidative stress, which plays a significant role in the development of AMD and DR [33,34]. 

One of the most widely used biomarkers in many studies is 8-OHdG, produced by the oxidative damage to DNA by reactive oxygen and nitrogen species, which serves as an established marker of oxidative stress. High levels of mitochondrial 8-OHdG have been correlated with increased mutation, deletion, and the loss of mtDNA, as well as apoptosis in H9C2 cardiac cells [35] and astrocytes [36]. 8-OHdG is increased in ARPE-19 cells under hydrogen peroxide exposure [19,37,38], in AMD [39,40,41] and RD serum patients [42], and in aged RPE-choroid mice [43]. This is consistent with our results, where we observed that H_2_O_2_ resulted in an 8-OHdG increase in ARPE-19 cells. We have shown the capacity of CoQ_10_ to restore DNA damage as similar results in our previous studies with vitamin D [38]. Similar results were obtained in a rat model of metabolic syndrome [44], where CoQ_10_ administration dose-dependently decreased the serum 8-OHdG levels in the control group and in healthy adult subjects supplemented with CoQ_10,_ where a delay of the formation of 8-OHdG in lymphocyte DNA was observed [45]. Fluorescence studies have demonstrated that ubiquinone homologues, CoQ_10_ included, possess a strong ordering effect on the lipid bilayer [46], and Tomasetti et al. hypothesized that the enrichment of human lymphocyte cells with ubiquinone-10 yielded an ordering and condensing effect on cell membranes, likely restricting the number of hydroxyl radicals which are capable of reaching cells’ DNA [47].

The potent protective and synergistic effects of CoQ_10_ and Nutrof were also corroborated by their efficacy in inhibiting the apoptosis of RPE cells reducing the levels of caspase-3 and TUNEL, since it has been demonstrated that the exposure of ARPE-19 cells to concentrations of H_2_O_2_ promotes apoptosis [37]. In this sense, CoQ_10_ with Nutrof enhances oxidative stability more efficiently than the sum of the individual antioxidant effects. Similar results were obtained in the literature by adding the CoQ_10_ complex in vitro and in vivo experiments in RPE under oxidative stress and other types of retinal cells such as RGCs [48]. 

The activity of antioxidant enzymes, among SOD2, which occurs in the mitochondrial matrix, and CAT [49] represents an important sign of the defense mechanism against ROS-induced oxidative stress [50]. A significant decrease in the mRNA expression of *SOD2* and *CAT* was observed in the H_2_O_2_ group when compared to the controls. In our study, neither CoQ_10_ nor N were able to restore this effect. In contrast, CoQ_10_ has been found to reduce the *SOD2* expression after an increase in the amount of enzymes following H_2_O_2_ application in astrocytes [51], RGCs [52,53] and retinal layers of porcine explants [54]. Moreover, in cancer progression, SOD2 has a dichotomous role [55]. These authors observed a reduction in Sod2-to-Gpx1 and Sod2-to-catalase ratios in DRP-TpoKO mice (follicular thyroid cancer model), indicating an inability to scavenge ROS. Furthermore, a stressful situation in age-related human granulosa cells in ovaries causes a decrease in *SOD2* and *CAT* mRNA and any relative proteins [56]. CoQ_10_ is well known to be a powerful nutritional supplement with antioxidant properties; however, it also exerts a protective role during inflammatory processes [11]. The anti-inflammatory effects of CoQ_10_ have already been corroborated through various clinical studies associated with chronic diseases, in particular, cardiovascular diseases, kidney disease, chronic obstructive pulmonary disease, non-alcoholic fatty liver disease, and neurodegenerative diseases [57]. For this reason, CoQ_10_ has been proposed as a possible adjuvant treatment in viral infections that causes a systemic inflammatory response [58]. In this sense, we investigated the potential role of CoQ_10_ and the nutritional complex in the downregulation of several inflammatory cytokines. Oxidative and inflammatory mediators, such as caspase 1, IL12p70, IL17A, IL18, IL1β, IL6, RANTES, and TNFα, play a vital role in the development of AMD [59,60,61,62,63,64,65] and DR diseases [66,67,68]. Hydrogen peroxide only induced a significant upregulation of both caspase-1 and RANTES; however, CoQ_10_ restored the caspase-1, TNFα, and IL-1β levels. It seems that, for some treatments, some cytokines are released earlier than for other treatments that are kept in the intracellular area longer when compared to the control group. The combination of both treatments used had no restorative effects. These results agree with the recent meta-analyses [69] that explain the role of the declining production of pro-inflammatory cytokines by inhibiting NF-κB gene expression, which is involved in the expression of pro-inflammatory cytokines, such as TNF-α [70,71]. In addition, inflammatory cytokines such as IL-1β were markedly decreased, and the expression of antioxidant genes (e.g., *SOD1*) was notably increased in ARPE-19 cells co-exposed to CoQ_10_ and H_2_O_2_ when compared to cells treated with H_2_O_2_ alone [15]. Interestingly, a study in human peripheral blood mononuclear cells cultured and pretreated with CoQ_10_ demonstrated that TNFα secretion was significantly decreased [72], but no changes in IL-1β were observed.

Mitochondrial dysfunction in RPE is one of the most important events observed in neovascular AMD patients [2,23,73,74], and it is often associated with a decrease in the mtDNA content in many disease with the overproduction of ROS in human RPE cells [75]. The oxidation of ARPE-19 cells induced the depletion of mtDNA as demonstrated by the decrease in the mtDNA on RPE cells. Our data show that CoQ_10_ combined with Nutrof prevents mtDNA release from the mitochondria to the cytosol and the circulation. Other studies in the skeletal muscle of mice described this effect [76]. However, there were no differences in the mtDNA content among the control or CoQ_10_-treated groups in ischemic retinas in a murine model [52]. Anion superoxide, as an estimation of ROS production, increased after hydrogen peroxide, and this was only improved by using both treatments together in our study. Cells with hydrogen peroxide were almost statistically significant when compared to the control, probably due to the sample size. A similar effect was found with idebenone, a quinone with similarities to the naturally occurring CoQ_10_. The treatment with idebenone significantly decreased the intracellular ROS formation [77] and ameliorated the cytotoxic effects of oxidative stress on RPE cells. In vivo investigations in age-related mice oocyte CoQ_10_ restored oocyte mitochondrial gene expression, improved mitochondrial activity [78]. Moreover, oxidative injury in rat pancreatic beta cells revealed the role of CoQ_10_ in reducing ROS levels [79]. 

Other mitochondrial components such as mtΔψ and mitochondrial membrane permeability (mPT) could be affected after oxidative stress and could initiate the degradative processes [80]. CoQ_10_ participates in the electron transport chain that takes place during aerobic cellular respiration in the mitochondria, meaning it is essential for the production of energy in cells [81,82]. In this sense, we found a beneficial effect of CoQ_10_ decreasing mtΔψ using a JC-1 marker. The effect was also observed but with less evidence in groups containing Nutrof. Consistent with our observations, studies have reported the same effect in ARPE-19 cells after chemical hypoxia. CoQ_10_ counteracted this phenomenon, significantly preventing mitochondrial membrane depolarization in more than 50% of ARPE-19 cells examined [48]. These authors described that CoQ_10_ is significantly more effective than other antioxidants (vitamin A, C, E) [83,84], and confer this effect to the participation of CoQ_10_ in complexes I and III of the respiratory chain with the mitochondrial permeability transition pore (mPTP); the association of ubiquinone Q_10_ with both complexes was in favor of this possibility, suggesting that CoQ_10_ could be part of the mPTP complex. In this sense, Zhong et al. proposed that the protective effect of CoQ_10_ might be associated with its role as a mobile electron transporter [85]. CoQ_10_ can correct the disorder of the electron transfer and improve the Q cycle, thus attenuating Ca^2+^ overload and cytrochome *c* release [47].

Mitochondrial dynamics is an essential process, and, in this study, we focused our attention on DRP-1 expression, known to be involved in the processes of fusion/fission and the energy regulation of the mitochondria. An abnormal activation of DRP-1 serves to eliminate damaged mitochondria during fission [18]. DRP-1 was altered after oxidative stress in ARPE-19 cells [86], in a murine model of long-term exposure to blue light, especially the ONL and RPE cells [87], in streptozotocin (STZ)-induced diabetic mice [88], and recently in a choroidal neovascularization (CNV) murine model, suggesting that mitochondrial fission in RPE contributes to angiogenesis development [89]. Our results indicated that CoQ_10_ in combination with Nutrof significantly decreased DRP-1, whereas H_2_O_2_ induced DRP-1 activation. In vitro studies have shown that CoQ_10_ prevented mitochondrial dynamic imbalance by reducing DRP-1 in murine neuronal HT22 cells [90] and other compounds, such as chrysoeriol, a flavonoid molecule, which protects ARPE-19 cells from oxidative stress through a decrease in DRP1 [84]. Interestingly, in vitro experiments with Drp1^−/−^ cells reveal that they are protected against apoptosis [91] and DRP-1 inhibition reduced the cleavage of caspase-3 and PARP in hepatocytes [8], suggesting that targeting DRP-1 may be protective against apoptosis.

In most markers studied, CoQ_10_ has slight antioxidant activity in human RPE cells exposed to oxidative stress by treatment with hydrogen peroxide; however, CoQ_10_ increases its beneficial activity with the nutritional complex, Nutrof (Table 1), providing a strong and synergistic effect in some cases. A possible explanation in this regard could be that CoQ_10_ is capable of regenerating other sources of antioxidants, such as high levels of NADPH quinone reductase, which has been postulated to produce the reduced form of CoQ_10_ in the epidermis, and it is necessary to reduce this from ubiquinone to ubiquinol in order for it to act as an antioxidant [92]. For all these functions, CoQ_10_ must be distributed among cell membranes, and that distribution seems to be regulated by specific proteins such as members of the UbiB family of atypical kinases/ATPases [46].

In particular, its effectiveness in reversing cellular damage and the consequent apoptosis is revealed when acting at a mitochondrial level. The CoQ_10_ levels decrease with age and in AMD and DR patients; therefore, the possibility of increasing the CoQ_10_ levels in different organs or tissues through dietary supplementation is necessary to standardize the indications for its use, composition, and dose. Two investigations have been conducted in AMD patients using CoQ_10_ as a dietary supplement [93,94]. The results have shown a slight improvement in visual function after treatment and a decrease in the area covered by drusen. 

In conclusion, our results suggest that adding CoQ_10_ to the Nutrof Total formula shows a synergistic effect when compared to the individual supplementation in scavenging, restoring, and/or preventing apoptosis and mitochondrial stress-related damage in RPE cells. These results suggest that adding CoQ_10_ could be a valid strategy for ameliorating early mitochondrial changes in degenerative processes such as AMD or DR. However, although the addition of CoQ_10_ to a nutritional complex seems to be promising to improve and prevent the progression of early and intermediate stages of AMD, additional research, mainly related to bioavailability, distribution, and interactions between antioxidant molecules, is necessary.

## 4. Materials and Methods

### 4.1. Cell Culture

Human retinal pigment epithelial cells (ARPE-19) were obtained from the American Type Culture Collection (ATCC) (CRL-2302, Manassas, VA, USA) and were grown to 70% confluency in Dulbecco’s modified Eagle’s medium (DMEM; D6429, Sigma-Aldrich, St. Louis, MO, USA) containing 10% fetal bovine serum (FBS; 10270106 Gibco ThermoFisher, Paisley, UK), 1% fungizone (Gibco, Carlsbad, CA, USA), and penicillin–streptomycin (Gibco, Carlsbad, CA, USA) in a 37 °C incubator with 5% CO_2_. The culture medium was replaced three times per week and split into the proper culture plate according to the subsequent experiments. After plating and reaching confluency, cells were maintained up to 2 months at 1% FBS, replacing the medium three times per week to reach the RPE phenotype until needed for the experiments as explained below.

### 4.2. Phenotypic Characterization by Flow Cytometry (FC) and Immunofluorescence

After reaching confluence in 24-well plates, the medium was changed to 1% FBS and replaced three times per week up to 2 months. To verify that ARPE-19 cells preserved their phenotype, RPE65 (ab231782, Abcam, Cambridge, MA, USA) and ZO1-Alexa Fluor-594 (339194, Invitrogen-Life Technologies, Carlsbad, CA, USA) were performed by FC (Figure S9A,B) and cytokeratin-18 (CK-18, M7010, DAKO, Santa Clara, CA, USA) antibodies were performed by immunofluorescence using CytoFLEX S (Beckman Coulter, Brea, CA, USA) (Figure S9C). Briefly, ARPE-19 cells were fixed with 4% of paraformaldehyde (PFA) for 10 min at 4 °C followed by three washes with FACS-Buffer (PBS 1X + 2% BSA + 5 mM EDTA). The cells were incubated in the dark for 30 min at RT with ZO1-Alexa Fluor-594 and RPE65 antibody prelabelled with FlexAble CoraLite^®^ Plus 555 Antibody Labeling Kit for Rabbit IgG (KFA002, Proteintech, Manchester, UK) according to the manufacturer’s instructions. After the incubation, the cells were washed 3 times with FACS-Buffer and were resuspended in 500 µL of FACS Buffer to measure the fluorescence. Data were analyzed with CytExpert software (Beckman Coulter, Brea, CA, USA). ARPE-19 cells (175,000 cells) were seeded on coverslips, and, after an experimental period of time, they were fixed with 4% of PFA in PBS for 10 min, washed with PBS, and labeled with active CK18 antibody diluted in blocking buffer containing 1% bovine serum albumin (BSA), 0.5% Triton X-100, 0.2% sodium azide, and 1% FBS overnight at 4 °C. Cells were incubated with the secondary fluorescent antibodies goat anti-mouse 488 (1:250, A11029, Life technologies, Gaithersburg, MD, USA) in blocking buffer for 1 h in the dark. Nuclei were labeled with 4′,6-diamidino-2-phenylindole (DAPI; Sigma-Aldrich, St. Louis, MO, USA). The morphology of cells was observed under a confocal microscope (LSM800, Zeiss, Oberkochen, Germany).

### 4.3. Cell Viability Determination after Oxidative Stress Induction and Treatments Application

The 3-(4,5-dimethylthiazol-2-yl)-2,5-diphenyltetrazolium bromide (MTT) reduction assay was used to determine cell viability using the CellTiter 96^®^ AQueous One Solution Cell Proliferation Assay (Promega, Madison, WI, USA) following the manufacturer’s instructions. Experiments were carried out on 96-well plates seeded with 32,000 ARPE-19 cells per well. Once cells were confluent, a culture medium was changed to 1% FBS and maintained for 2 months. In order to select the appropriate and safe doses for the efficacy experiments, we evaluated ten doses of CoQ_10_ (0.01, 0.05, 0.1, 0.25, 0.5, 1, 2, 10, 50, 100 µM) and H_2_O_2_ (100, 200, 400, 600, 800, 1000, 1200, 1400, 1600, 1800, 2400 µM) at different timepoints (CoQ_10_: 1, 2, and 4 h; H_2_O_2_: 2, 6, and 24 h). Moreover, five doses of the N (0.01, 0.04, 0.07, 0.14, 0.70 mg/mL) and N + CoQ_10_ treatments (NQ; 0.1, 0.5, 1, 2, 10 µM CoQ_10_) were tested at three different timepoints (1, 2, and 4 h) on ARPE-19 cells (passages p8-p14). Then, samples were subjected to the cell viability test according to the manufacturer’s instructions. Results were obtained by reading the 492 nm absorbance using a Sunrise-basic Microplate reader (Tecan Austria GmbH, Grödig, Austria) and are shown in Figures S6–S8. The control group consisted of the solution used to dissolve the treatments, namely acetone 0.002% in cell culture media. 

### 4.4. Selection of the Oxidative Stress Conditions and Treatment Concentrations

According to the results obtained (Figures S6 and S7), H_2_O_2_ (Panreac, Barcelona, Spain) at 600–800 µM were selected as the safe pro-oxidant stimulus to induce RPE oxidative stress. CoQ_10_ (synthetic origin, provided by Thea Laboratoires, Clermont-Ferrand, France) at 0.1 µM, Nutrof Total^®^ (N; 0.01 mg/mL, see Table S2 for composition; Thea Laboratoires) or Nutrof Total^®^ plus CoQ_10_ (NQ) at a total equivalent concentration of 62.34 µg/mL (Figure S8) was selected. This concentration was used for both the Q and NQ treatments in our experiments in order to have consistency in our comparisons between the Q and NQ treatments, and these concentrations were found to be non-toxic for ARPE-19 cells. Treatments were added in concomitance with the oxidative damage, as shown in Table S1.

### 4.5. Cell Apoptosis Evaluation by TUNEL and Caspase-3 Immunofluorescence

The apoptotic stage of the cells was also evaluated, using caspase-3 as a marker of early stage apoptosis and TUNEL as a marker of late stage apoptosis. For the TUNEL assay, an in situ cell death detection kit with TMR Red was used following the manufacturer’s instructions (12156792910, Roche, West Sussex, UK). ARPE-19 cells (175,000 cells) were seeded on coverslips, and after experimental procedures, they were fixed with 4% of PFA in PBS for 10 min, washed with PBS, and labeled with active caspase-3 antibodies (1:100, G7481; Promega, Madison, WI, USA) diluted in blocking buffer containing 1% bovine serum albumin (BSA), 0.5% Triton X-100, 0.2% sodium azide, and 1% FBS overnight at 4 °C. Cells were incubated with the secondary fluorescent antibodies goat anti-mouse 488 (1:250, A11029, Life technologies, Gaithersburg, MD, USA) in blocking buffer for 1 h in the dark. Nuclei were labeled with 4′,6-diamidino-2-phenylindole (DAPI; Sigma-Aldrich, St. Louis, MO, USA). The morphology of cells was observed under a confocal microscope (LSM800, Zeiss, Oberkochen, Germany). 

### 4.6. Analysis of Mitochondrial Function 

#### 4.6.1. Analysis for Membrane Mitochondrial Potential (mtΔψ)

The MtΔψ status was performed in live ARPE-19 cells using the membrane-permeant JC-1 (5,5’,6,6’-tetrachloro-1,1’,3,3’-tetraethylbenzimidazolylcarbocyanine iodide) dye, which is widely used in apoptosis studies to monitor mitochondrial health. JC-1 is a ratiometric dye that forms aggregates in highly polarized/energized mitochondria and emits an orange-red fluorescence at 595 nm (red/phycoerythrin). In depolarized mitochondria, JC-1 remains as monomers and emits green fluorescence at 530 nm (green/fluorescein isothiocyanate). After culturing 175,000 cells on a 10 mm dish (Menzel-Glaser, Waltham, MA, USA), they were incubated with JC-1 (2.5 µM) for 15 min in the dark at 37 °C according to the manufacturer’s instructions (T3168, Invitrogen, Molecular Probes, Inc, Eugene, OR, USA). Three passages were analyzed and images were taken under a confocal fluorescence microscope (LSM800, Zeiss, Oberkochen, Germany) at ×40 magnification. Relative levels of the intensities of the monomers/aggregates of JC-1 fluorescence were quantified using Fiji/ImageJ, an open-source Java-based image analysis software (NIH, Bethesda, MD, USA). 

#### 4.6.2. Detection of Mitochondrial Superoxide Production Using MitoSOX

ARPE-19 cells seeded on a 10 mm dish (175,000 cells per dish) (Menzel-Glaser, Waltham, MA, USA) were stained with MitoSOX Red mitochondrial superoxide indicator for live-cell imaging (M36008, Molecular Probes Inc, Eugene, OR, USA) (*n* = 3). Briefly, the MitoSOX component was dissolved in dimethyl sulfoxide (DMSO) in a medium without FBS to produce the mitoSOX reagent working solution in which the cells were incubated for 10 min at 37 °C and protected from light. Then, they were gently washed three times with PBS for 10 min. Finally, images were taken under a confocal fluorescence microscope (LSM800, Zeiss, Oberkochen, Germany) at ×40 magnification. 

#### 4.6.3. DRP-1 Immunofluorescence

ARPE-19 cells were plated in 96-well plates for 2 months as explained above, and after the experimental procedures, they were fixed with 4% of PFA in PBS for 10 min, washed with PBS three times, and permeabilized with blocking buffer for 10 min at 4 °C. Then, cells were incubated with the rabbit polyclonal anti-DRP-1 (1:250 dilution, ab184247, Abcam, Cambridge, MA, USA) antibody and subsequently with goat anti-rabbit Alexa fluor 594 (1:250, A-11012, Thermo Fisher Scientific, Paisley, UK). Nuclei were stained with DAPI (Sigma-Aldrich, St. Louis, MO, USA). Cells were analyzed under a confocal microscope (LSM800, Zeiss, Oberkochen, Germany) at ×40 magnification, and the intensity of fluorescence was measured using a home-made plugin tool developed for Fiji/ImageJ, an open-source Java-based image analysis software. The plugin was developed by the Imaging Platform of the CIMA Universidad de Navarra.

#### 4.6.4. Mitochondrial DNA Amount (mtDNA)

DNA extraction was performed using the DNeasy Blood & tissue extraction kit (Qiagen, Hilden, Germany). All possible RNA was digested by a RNAse reaction. The extracted DNA was measured by spectrophotometry with the ND-1000 Spectrophotometer (Nanodrop, Thermo Fisher Scientific, Waltham, MA, USA) to check both the final concentration and DNA quality. The expression of *12S* was measured using a 7300 Real Time PCR System (Applied Biosystems; Life Technologies, Carlsbad, CA, USA) and the Taqman Assays 12S Hs02596859_g1 (Applied Biosystems; Life Technologies, Carlsbad, CA, USA). For relative calculations, we compared the Ct results of treated samples vs. the control samples in a straight pattern of decreasing concentrations of mtDNA.

### 4.7. Measurement of 8-Hydroxidioxiguanosine (8-OHdG) under Oxidative Stress Conditions

Oxidative damage was measured in the DNA of ARPE-19 subjected to H_2_O_2_ for 1 h, and antioxidant treatments were added in concomitance for 30 min. To evaluate the effect of antioxidant treatments, we added 0.1 µM of CoQ_10_ and/or 0.01 mg/mL of Nutrof to the media. Three hundred ng of DNA was evaluated using the EpiQuik TM 8-OHdG DNA Damage Quantification Direct kit #P-60003 (Epigentek, Farmingdale, NY, USA). Data are presented in % 8-OHdG vs. control.

### 4.8. DNA Multiplex Cytokine Analysis 

Samples were subjected to H_2_O_2_ for 2 h (Table S1), and treatments were added in concomitance for 1 h. Then, ARPE-19 lysates and supernatants were collected, and the following cytokines levels were measured using the ELLA multiplex platform (Biotechne, Minnesota, MN, USA): Caspase 1, IL12-p70, IL17A, IL18, IL1β, IL6, RANTES, and TNFα. Cell lysates were obtained by collecting cells using tripsin and adding a lysis buffer. Then, samples were centrifuged at 13,000 rpm for 20 min, pellets were discarded, and supernatants were used to determine the intracellular cytokines’ levels. 

### 4.9. RNA Analysis: Expression of IL-1β, SOD2, and CAT

Samples were subjected to H_2_O_2_ for 1 or 2 h (Table S1) and antioxidant treatments were added in concomitance for 30 min or 1 h, respectively. Then, ARPE-19 lysates were collected, and the subsequent methods were performed. RNA extraction was performed using the Illustra^TM^ RNAspin extraction kit (GE Healthcare, Chicago, IL, USA). All possible DNA was digested by the DNAse reaction. The extracted RNA was measured by spectrophotometry with the ND-1000 Spectrophotometer (Nanodrop, Thermo Fisher Scientific, Waltham, MA, USA) to check both the final concentration and RNA quality. The reverse transcription of 500 ng of RNA from each sample was performed in a total of 20 µL of reaction with PCR Retrotrancription System (Quantabio, Beverly, MA, USA) under the following conditions: 25 °C—5 min; 42 °C—30 min; 85 °C—5 min. The expression of genes was measured using a 7300 Real Time PCR System (Applied Biosystems; Life Technologies, Carlsbad, CA, USA) and the Taqman Assays *SOD2* Hs00167309_m1, *IL1β* Hs01555410_m1, and *CAT* Hs00156308_m1. *18S* and *GAPDH* genes (Hs99999901_s1 and Hs99999905_m1, respectively, Applied Biosystems; Life Technologies, Carlsbad, CA, USA) were used for normalization purposes. For relative calculation, we compared the Ct results of the *SOD2*, *IL1β*, and *CAT* expression of the control samples vs. antioxidants.

### 4.10. Statistical Analysis

For quantitative variables, the Shapiro–Wilk normality test was applied and all parameters were subjected to the one-way analysis of variance (ANOVA) or Kruskal–Wallis followed by the Bonferroni post hoc test. All groups were normalized by each pass and compared against a control group. Data are expressed as mean ± SEM. A difference of *p* < 0.05 was considered statistically significant. GraphPad Prism 8.0 (GraphPad Prism Software Inc., San Diego, CA, USA) was used for statistical analysis. 

## Figures and Tables

**Table 1:** Graphical summary showing the protective effects of CoQ_10_ on human RPE damaged by H_2_O_2_.

Processes	Markers	H_2_O_2_-RPE Cells	Antioxidant Treatment + H_2_O_2_		
			CoQ10	Nutrof	N + CoQ10
Oxidative and DNA stress	8-OHdG	↑ *	-	-	*p =* 0.055
	*SOD2*	↓ *2 h	-	-	-
	*CAT*	↓ *1 h	-	-	-
Apoptosis	Caspase-3	↑ ***	-	*	**
	TUNEL	↑ ***	*	-	***
Inflammation	TNF-α	Unchanged supernatants	*	-	-
		↓ Lysate	*	-	-
	Caspase-1	Unchanged supernatants	-	-	-
		↑ Lysate	*	-	-
	*ILβ1*	↓ **2 h	*	*	-
	RANTES	↑ Lysate	-	-	-
	IL6, IL17A, IL18	-	-	-	-
	IL12p70	↓ Lysate	*p =* 0.0634	-	-
Mitochondrial dysfunction	MitoSOX	↑ *	-	-	*p =* 0.053
	mtDNA	↑ *p =* 0.069	-	-	*
	JC-1	↑	*	-	-
	DRP-1	↑ *	-	-	*

Oxidative damage was induced by H_2_O_2_ and treated with CoQ_10_ (Q), Nutrof (N), and CoQ_10_ plus Nutrof (NQ). The damage caused changes in DNA, gene expression, apoptosis, increases in several inflammation markers (ILs), and alterations to mtDNA and mitochondrial functions. Furthermore, the antioxidants together with NQ were able to protect RPE cells from oxidative stress by decreasing the apoptosis and recovering mtDNA and DRP-1 levels. * *p* < 0.05, ** *p* < 0.01 and *** *p* < 0.001.

## Data Availability

All data are available within the manuscript and upon request to corresponding authors.

## Supplementary Materials

The following supporting information can be downloaded at https://www.mdpi.com/article/10.3390/ijms25158070/s1.
