# Supplementary material for: The Scavenging Activity of Coenzyme Q10 Plus a Nutritional Complex on Human Retinal Pigment Epithelial Cells
Source: Int J Mol Sci. 2024 Jul 24;25(15):8070. doi: 10.3390/ijms25158070 (PMC11311961; doi:10.3390/ijms25158070)
Supplement: Supplementary file 1 [file ijms-25-08070-s001.zip › ijms-3061224-supplementary.pdf]

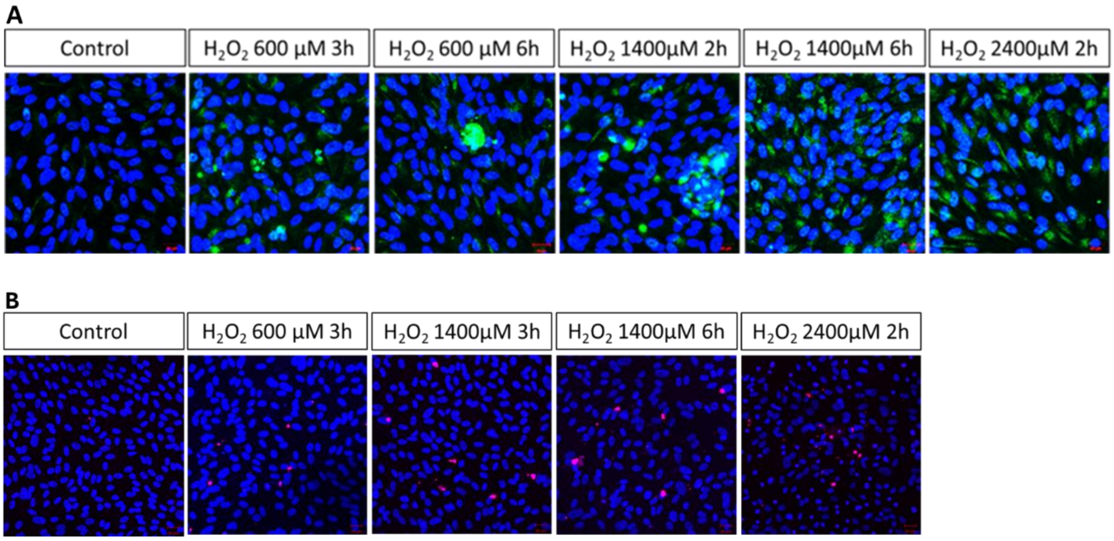

Figure S1. Early apoptosis observed by caspase-3 immunofluorescence (green) (A) and TUNEL assay (red) (B) in ARPE-19 cells to establish experimental conditions for subsequent analysis (n=3). The conditions were control (without H_2O_2), H_2O_2 at different concentrations (600, 1400 and 2400 µM for 2, 3 and 6 h). Nuclei were labeled with 4',6-diamidino-2-phenylindole (DAPI) (blue). Scale bar: 20 µm.

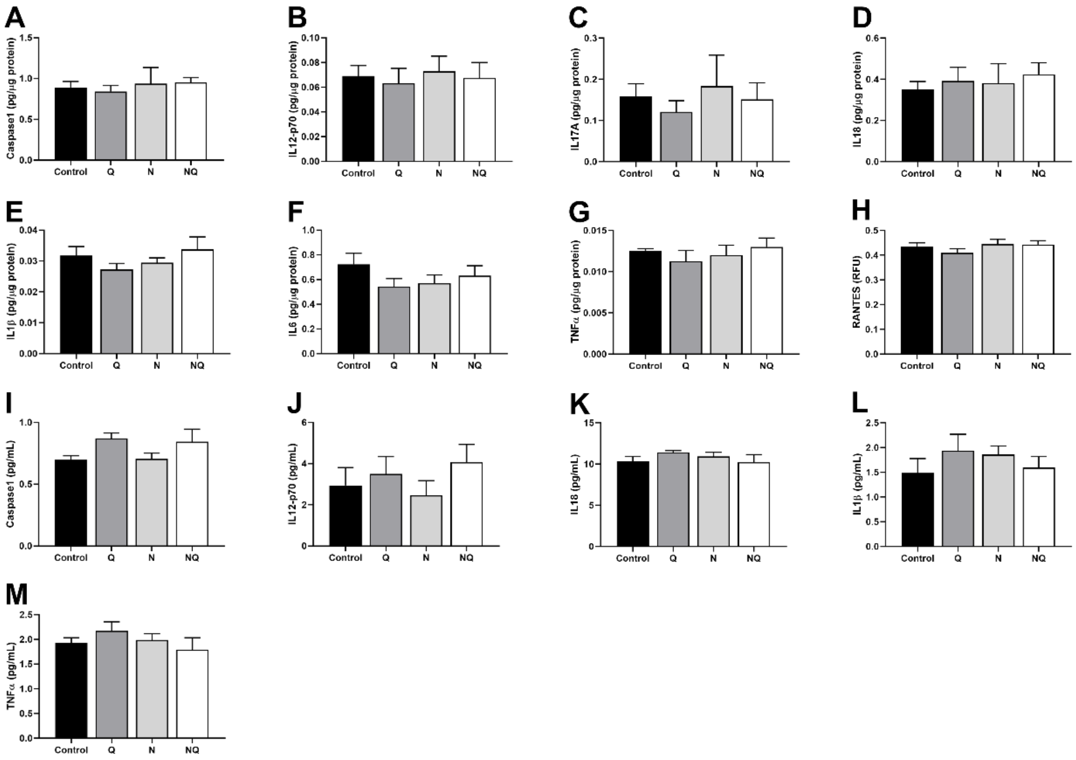

Figure S2. Quantification of cytokine levels of caspase 1 (A), IL12p70 (B), IL17A (C), IL18 (D), IL1β (E), IL6 (F), TNFα (G) and RANTES (H) levels in ARPE-19 cells lysates and caspase 1 (I), IL12p70 (J), IL18 (K), IL1β (L) and TNFα (M) supernatants with different treatments under basal conditions (n=4). Lysates' data are presented as pg/µg protein and supernatants' data are presented as pg/µg protein. RANTES data are presented as RFU (relative fluorescence units). Q-coenzyme Q_{10}, N-Nutrof total, NQ-Nutrof total + CoQ_{10}.

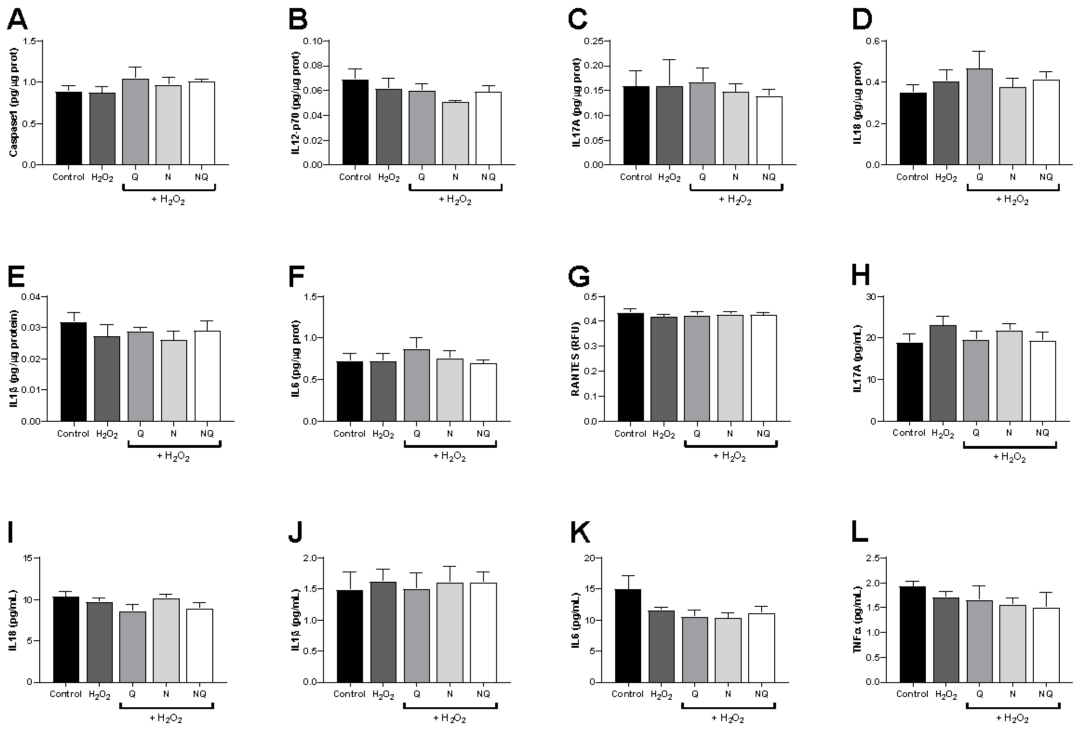

Figure S3. Quantification of cytokine levels of caspase 1 (A), IL12p70 (B), IL17A (C), IL18 (D), IL1β (E), IL6 (F), RANTES (G) and IL17A (H) levels in ARPE-19 cells lysates (A-G) and supernatants (H-L) of IL17A (H), IL18 (I), IL1β (J), IL6 (K) and TNFα (L) with different treatments under oxidative stress conditions (n=4). Lysates' data are presented as pg/µg protein and supernatants' data are presented as pg/µg protein. RANTES data are presented as RFU (relative fluorescence units). Q-coenzyme Q_{10}, N-Nutrof total, NQ-Nutrof total + CoQ_{10}.

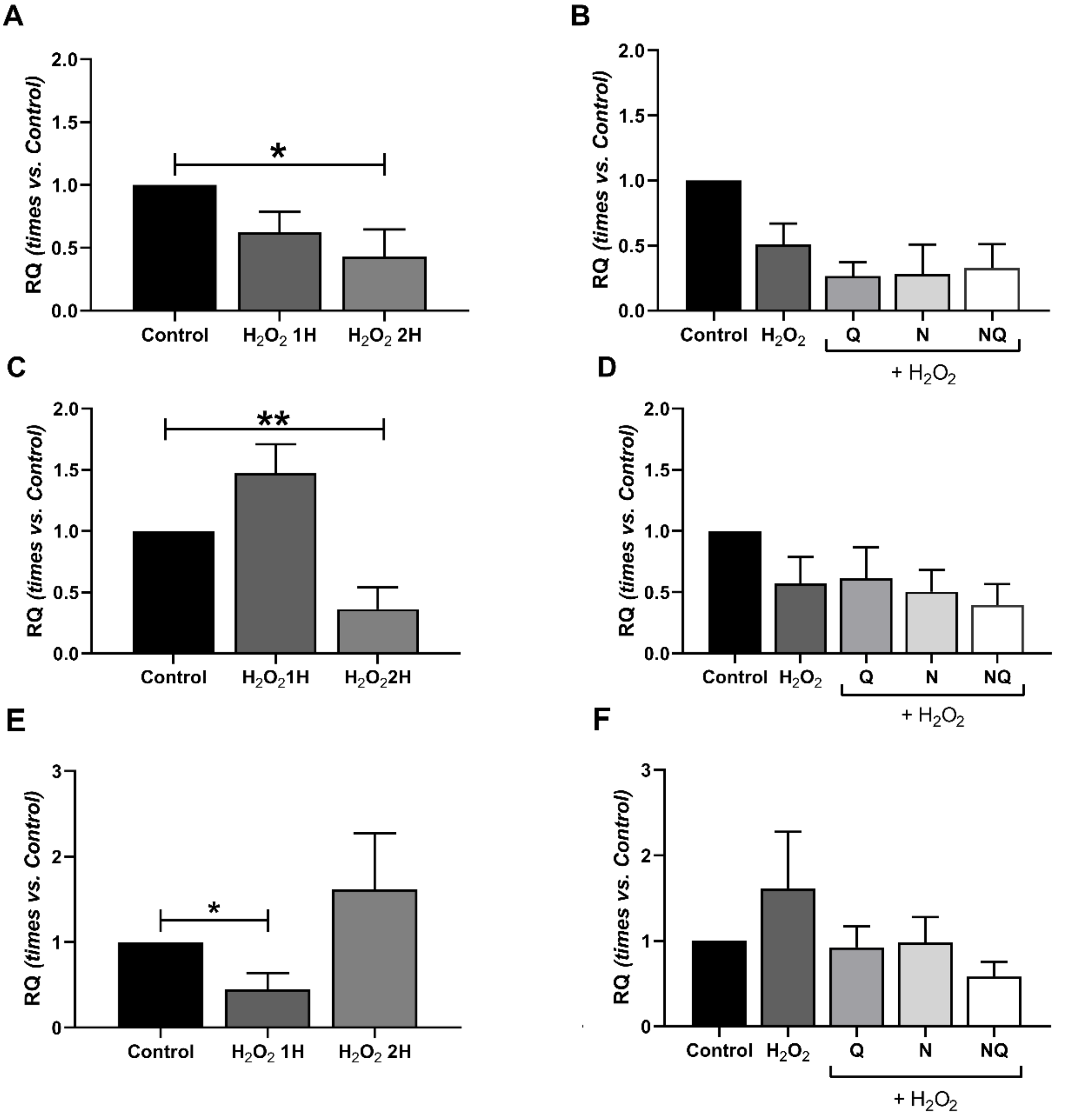

Figure S4. *SOD2* expression in ARPE-19 cells with 1 and 2 h of damage with H_2O_2 compared to control group (A). *SOD2* expression in ARPE-19 cells with 1 h of H_2O_2 in concomitance with treatments vs. H_2O_2 group (B) (n=4). *ILβ1* expression in ARPE-19 cells with 1 and 2 h of damage with H_2O_2 compared to control group (C) and *ILβ1* expression in ARPE-19 cells with 2 h of H_2O_2 concomitance with treatments vs. H_2O_2 group (D). *CAT* expression in ARPE-19 cells with 1 and 2 h of damage with H_2O_2 compared to control group (E) and *CAT* expression in ARPE-19 cells with 2 h of H_2O_2 concomitance with treatments vs. H_2O_2 group (F). For all data mean ± SEM are presented. ** $p<0.01$ and *$p<0.05$ vs saline. Q-coenzyme Q_{10}, N-Nutrof total, NQ-Nutrof total + CoQ_{10}

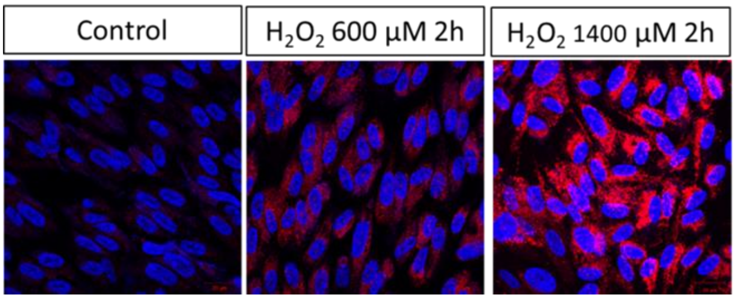

Figure S5. Mitochondrial superoxide indicator in ARPE-19 cells measured by MitoSOX (red) under confocal microscopy to establish experimental conditions for H_2O_2 to induce a dose dependent superoxide release (n=3). Nuclei were labeled with 4′,6-diamidino-2-phenylindole (DAPI) (blue). Scale bar: 20µm.

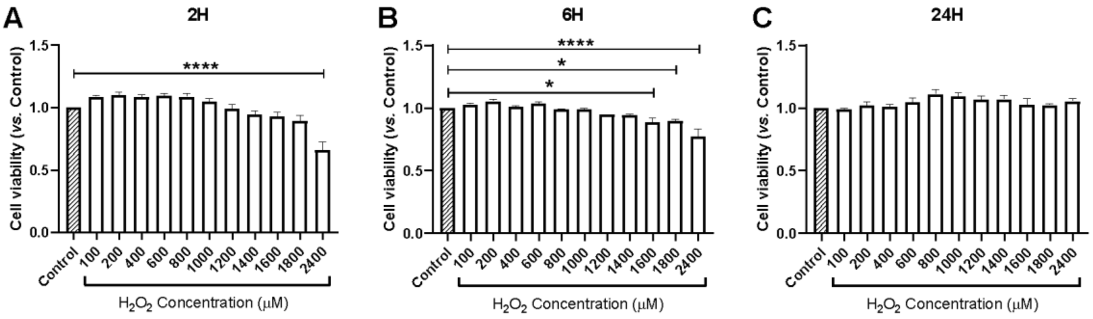

Figure S6. Cell viability measured by MTT in ARPE-19 cells for H_2O_2 at different concentrations and timepoints (n=3). * $p<0.05$ and **** $p<0.001$ vs. control. One-way ANOVA. C-control.

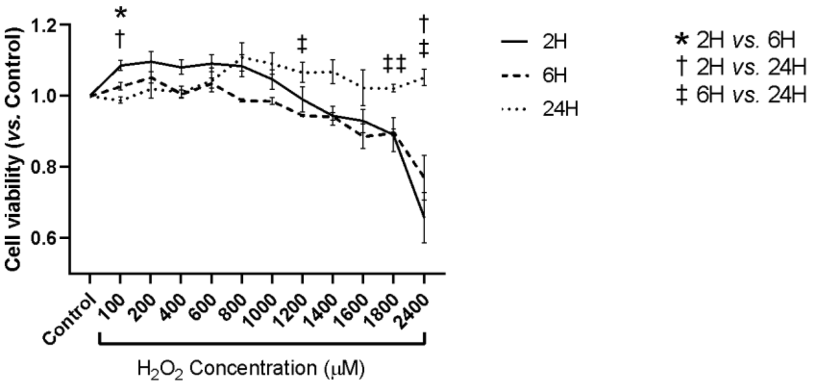

Figure S7. Cell viability measured by MTT in ARPE-19 cells after H_2O_2 addition at different concentrations and timepoints (n=3). Two-criteria ANOVA. Legend shows the statistical comparisons detailed in the graph.

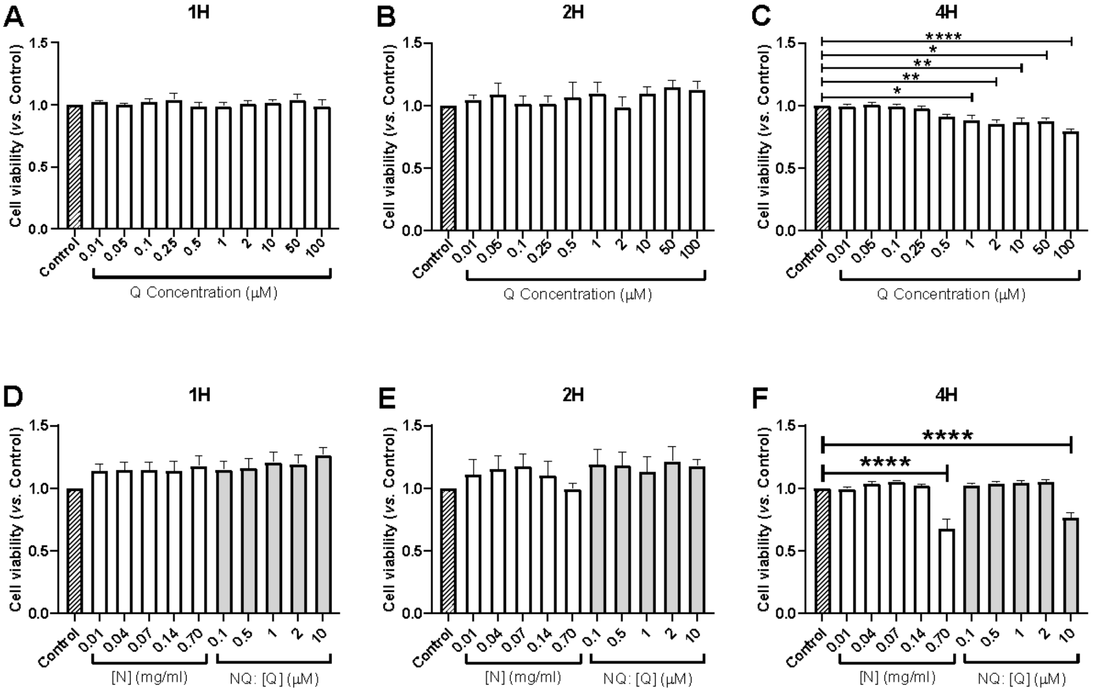

Figure S8. Cell viability measured in ARPE-19 cells for Q, N and NQ treatments at different concentrations and timepoints (n=3). * p<0.05, ** p<0.01, **** p<0.0001 vs. Control. C: control; Q: coenzyme Q_{10}; N: Nutrof total; NQ: Nutrof total + coenzyme Q_{10}.

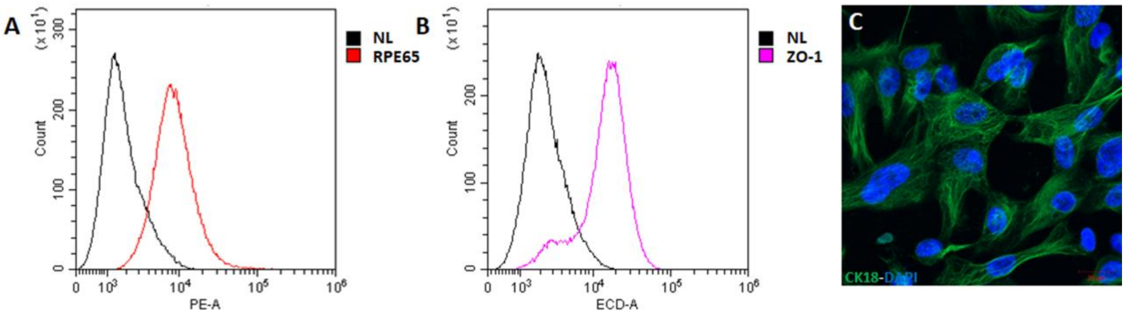

Figure S9. Graphics showed the phenotypic characterization of ARPE-19 cells by flow cytometry (RPE65 and ZO1 antibodies) (A, B respectively) and immunofluorescence with anti-CK18 antibody (green) in coverslips (C) (n=3). Nuclei were labeled with 4′,6-diamidino-2-phenylindole (DAPI) (blue). NL: non label. Scale bar: 20µm.

	$[H_2O_2]$	Total time	Q, N or NQ concomitance
**Caspase-3**	600 µM	3h	1h
**TUNEL**	600 µM	3h	1h
**JC-1**	800 µM	4h	1h
**MitoSOX**	600 µM	2h	1h
**DRP1**	600 µM	2h	1h
**8-OHdG**	600 µM	1	30 min
**Cytokines**	600 µM	2h	1h
**Gene expression**	600 µM	1 and 2h	30 min and 1h
**Mitochondrial DNA**	600 µM	2h	1h

Table S1. Setting up of experimental parameters (damage concentration and time, and concomitance with antioxidant treatments) used for each determination.

	**Nutrof Total® (mg)**
Vitamin C	60
Vitamin E	10
Vitamin D3	0.005
Zinc	10
Selenium	0.025
Copper	0.5
Lutein	10
Zeaxanthin	2
Fish oil	330
with	*132 EPA*<br>*66 DHA*
Glutathione	1
Resveratrol	1

Table S2**.** Composition of Nutrof Total® used in the study (per one capsule).
